# Polymyxin B and the Pulmonary Peril: A Case Series of Acute Respiratory Failure

**DOI:** 10.1155/crcc/5569011

**Published:** 2026-09-21

**Authors:** Lovely Thomas, John Victor Peter, Binu Susan Mathew, Sara Jose, Lisa Merin Joseph, Jeethu Joseph Eapen, Sirish Chandra Srinath Patloori, Binila Chacko

**Affiliations:** ^1^ Department of Critical Care, Christian Medical College, Vellore, India, cmch-vellore.edu; ^2^ Department of Clinical Pharmacology, Christian Medical College, Vellore, India, cmch-vellore.edu; ^3^ Department of Nephrology, Christian Medical College, Vellore, India, cmch-vellore.edu; ^4^ Department of Cardiology, Christian Medical College, Vellore, India, cmch-vellore.edu

**Keywords:** critically ill, hypercapnic, polymyxin B, respiratory failure, toxicity

## Abstract

**Background:**

The neurotoxicity potential of polymyxin B is underrecognized.

**Results:**

Four critically ill patients treated with polymyxin B for suspected or confirmed nosocomial Gram‐negative infections developed acute hypercapnic respiratory failure temporally associated with polymyxin B administration. Respiratory support escalated from room air or low‐flow oxygen to noninvasive or invasive mechanical ventilation after one, two, five, and seven doses of polymyxin. No alternative explanation for the abrupt deterioration was identified after clinical evaluation. Following cessation of polymyxin (*n* = 3) or dose reduction (*n* = 1), all patients were liberated from ventilation within 60 h. Measured polymyxin concentrations were above the therapeutic range in all patients; however, the delayed timing of sampling, measured at variable intervals after the respiratory events, limits conclusions regarding causality and dose‐dependent toxicity.

**Conclusion:**

Polymyxin B should be considered as a potential cause of acute hypercapnic respiratory failure in critically ill patients when no alternative explanation is apparent. Both idiosyncratic susceptibility and drug accumulation may contribute, although the delayed timing of drug concentration measurements precludes firm conclusions regarding dose‐dependent toxicity. Further prospective studies are required to define the mechanisms and risk factors.

## 1. Introduction

Polymyxins, discovered in the 1940s, were introduced into clinical use before modern drug development protocols were established. The intravenous use of polymyxins declined significantly by the 1970s, primarily due to concerns regarding their toxicity, particularly nephrotoxicity [1]. Although neurotoxicity is less frequently reported, polymyxins have been associated with adverse neurological effects, including dizziness, muscle weakness, facial and peripheral paraesthesia, vertigo, visual disturbances, confusion, ataxia, and neuromuscular blockade [2, 3]. Both nephrotoxic and neurotoxic effects are considered dose‐dependent and are generally reversible upon early discontinuation of therapy [4].

With the rise of multidrug‐resistant (MDR) Gram‐negative infections in the 1990s, colistin and polymyxin B regained importance as essential therapeutic options, especially in critically ill patients for whom few alternatives remained [5]. In this case series, we describe four critically ill patients admitted to the intensive care unit (ICU) in October and November 2024 who developed acute respiratory failure after initiating intravenous polymyxin B. We describe the temporal profile of these patients and postulate the mechanisms contributing to this phenomenon.

### 1.1. Ethics Statement

This retrospective case series was approved by the Institutional Review Board/Ethics Committee of Christian Medical College, Vellore (IRB Min No. 2502148 dated February 26, 2025). The requirement for informed consent was waived because the study involved retrospective review of anonymized patient data and posed minimal risk to participants.

## 2. Case Presentation

Four patients (Table 1) aged between 48 and 68 years received standard polymyxin B dosing: loading dose of 2.0–2.5 mg/kg (20,000–25,000 IU/kg) over 1 h followed by maintenance dose (MD) of 1.25–1.5 mg/kg (12,500–15,000 IU/kg) every 12 h, given as an infusion over 1 h. All four patients experienced new rapid onset Type II respiratory failure after one, two, five, and seven doses of polymyxin. Polymyxin B concentrations, measured using the liquid chromatography mass spectrometry, 3–8 days after symptom onset, were supratherapeutic. Symptoms resolved after cessation of the drug or reduction in the dose.

**Table 1:** Patient demographics, renal function, and outcomes.

	1	2	3	4
Age	66 years	49 years	48 years	68 years
Gender	Male	Female	Male	Male
Diagnosis	Diffuse large B cell lymphoma, chronic AF, post MVR (2016)	Emphysematous pyelonephritis, acute on CKD (on MHD)	Myelodysplastic syndrome, post chemotherapy	Nonischemic DCMY acute on CKD (no MHD)
APACHE II (predicted mortality)	20 (35.5%)	21 (38.9%)	17 (26.2%)	27 (60.5%)
Indication for polymyxin B	Suspected sepsis in immunocompromised host	Probable superadded nosocomial sepsis	Immunocompromised host with septic shock	*E. coli* bacteraemia (CRO)
Respiratory support prior to polymyxin therapy	Room air	Room air	Venturi mask 31%	2 L NP
Number of doses received prior to the first escalation of respiratory support	1 dose (LD)	7 doses (LD + 6 MD)	2 doses (LD + 1 MD)	5 doses (LD + 4 MD)
Clinical presentation prior to escalation of respiratory support	Increased work of breathing, hypercapnic respiratory failure	Increased drowsiness, hypercapnic respiratory failure	Sudden breathlessness, increasing drowsiness, hypercapnic respiratory failure	Worsening hypercapnic respiratory failure on NIV with drop in GCS
Respiratory support escalated to	NIV followed by intubation	Intubated	Intubated	NIV followed by intubation
Cardiovascular support prior to polymyxin therapy	Nor‐adrenaline—4 *μ*g/min	Nor‐adrenaline—4 *μ*g/min	Nor‐adrenaline—10 *μ*g/min	Nor‐adrenaline—5 *μ*g/min
Cumulative dose received prior to intubation (million units)	4.5 MU (LD + 4 MD)	6.0 MU (LD + 6 MD)	2.25 MU (*L* *D* + 1 MD)	9MU (LD + 10 MD)
Serum creatinine on day of initiation	1.03 mg/dL	2.4 mg/dL	1.26 mg/dL	2.05 mg/dL
Serum creatinine on day of intubation	1.20 mg/dL	3.4 mg/dL	1.64 mg/dL	2.37 mg/dL
Number of doses prior to sending serum polymyxin B level	9 doses (LD + 8 MD)	18 doses (LD + 17 MD)	6 doses (LD + 5 MD)	18 doses (LD + 17 MD)
Plasma polymyxin concentration	AUC_ss,24 h_ = 127.9 mg.hr/L	AUC_ss,24 h_ = 108.5 mg.hr/L	Average steady state plasma concentration = 4.8 ug/mL	AUC_ss,24 h_ = 131.6 mg.hr/L
Action taken	Stopped after 10 MD	Stopped after 18 MD	Stopped after 10 MD	Decreased to 5 LU and continued till completion of course
Duration of IMV	3 days	6 days	2 days;	8 days
Liberation from the ventilator	24 h–ventilatory requirements minimal; 48 h–cuff leak absent despite minimal ventilatory support; 60 h–successfully extubated.	After 15th MD–extubated; after 16th MD–recurrence of hypercapnic respiratory failure; NIV required for 5 h; following polymyxin B cessation–transitioned to room air after 5 h.	After 4th MD–extubated; after 5th MD–re‐intubation; following polymyxin B cessation–successfully extubated to Venturi mask within 24 h.	24 h after reducing the dose–extubated to NIV.
Additional points	No dialysis	Dialyzed after LD and 14th MD	No dialysis	No dialysis

*Note:* AUC_ss,24 h_ is reported for Patients 1, 2, and 4. For Patient 3, only the average steady‐state plasma concentration could be estimated because PK sampling was incomplete; it is not directly comparable with AUC_ss,24 h_.

Abbreviations: AF, atrial fibrillation; AUC, area under the curve; CKD, chronic kidney disease; CRO, carbapenem resistant organism; DCMY, dilated cardiomyopathy;GCS, Glasgow Coma Scale; IMV, invasive mechanical ventilation; LD, loading dose; MD, maintenance dose; MHD, maintenance haemodialysis; MU, million units; MVR, mitral valve replacement; RA, room air; VM, Venturi mask.

### 2.1. Patient 1

Within 5 h of receiving the polymyxin B loading dose, a 66‐year‐old man with diffuse large B‐cell lymphoma experienced acute respiratory acidemia necessitating noninvasive ventilation (NIV). Subsequent doses were followed by worsening hypercapnia, which culminated in intubation following the fourth MD. Neurological evaluation and imaging were unremarkable. Plasma polymyxin B concentrations, done after the eighth MD, were elevated (24‐h plasma AUC at steady state (AUC_ss,24 h_) AUC 127.9 mg.hr/L; recommended therapeutic range 50–100 mg·hr/L). Although ventilatory requirements were minimal within 24 h of drug discontinuation, extubation was delayed until 60 h because of an absent cuff leak, raising concern for upper airway edema and the risk of postextubation stridor. Extubation was successfully performed after the return of a positive cuff leak.

### 2.2. Patient 2

Polymyxin B was administered to a 49‐year‐old woman who was diagnosed with emphysematous pyelonephritis and septic shock. At 3 h following her sixth MD, she developed acute hypercapnic respiratory failure and a drop in Glasgow Coma Scale (GCS), necessitating intubation. Ventilatory requirements were minimal following intubation; however, extubation occurred only after the 15th MD due to difficulty in weaning. Magnetic resonance imaging (MRI) of the brain, electroencephalography (EEG), and cerebrospinal fluid (CSF) analysis were unremarkable. The suspicion of polymyxin toxicity was heightened by the recurrence of hypercapnic respiratory failure following the 16th MD. Polymyxin B levels were obtained approximately 36 h after the last hemodialysis session, with no intervening dialysis performed prior to sampling. Plasma polymyxin B concentrations were elevated (AUC_ss,24 h_ 108.5 mg.hr/L). Although polymyxin B is generally believed to undergo minimal dialytic clearance due to its high protein binding, the impact of different dialysis modalities on polymyxin pharmacokinetics has not been fully elucidated. The episodes of respiratory acidosis did not recur after its cessation.

### 2.3. Patient 3

Breathlessness and acute respiratory acidosis developed in a 48‐year‐old man with myelodysplastic syndrome and sepsis. At 2 h after the first maintenance dose, he complained that he “could not breathe” and developed acute respiratory acidosis necessitating intubation. There was no angioedema, stridor, or wheeze to suggest anaphylaxis or bronchospasm as alternative explanations for the acute respiratory deterioration. He was rapidly weaned off the ventilator and extubated after 36 h. A similar episode occurred after the fifth MD, necessitating reintubation. The average of plasma polymyxin B concentration was supratherapeutic at 4.8 *μ*g/mL (reference range of C_ss,avg_ 2–4 *μ*g/mL). As all the sample collection time points did not align precisely with the predefined schedule, the AUC_ss,24 h_ was not reported. The antibiotic was subsequently discontinued, and within 24 h, the patient was successfully extubated.

### 2.4. Patient 4

A 68‐year‐old man with chronic kidney disease, *E. coli* bacteraemia, and dilated cardiomyopathy developed respiratory failure necessitating NIV after the fourth MD and intubation after his 10th MD. There was a suspicion of polymyxin B toxicity in view of difficulty in liberation from the ventilator. Polymyxin B concentrations were elevated (AUC_ss,24 h_ = 131.6 mg.hr/L). Following dose reduction, he was extubated within 24 h.

**Figure 1:**
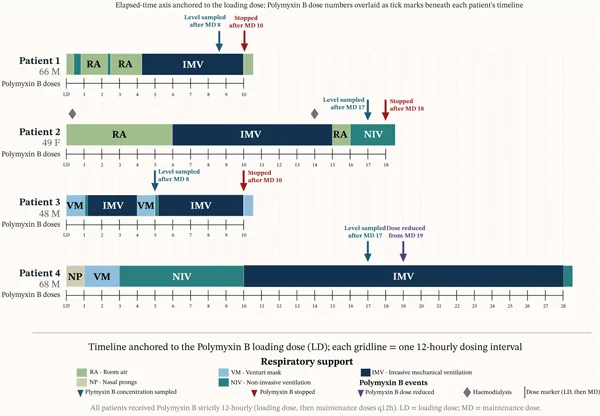
Temporal profile of respiratory support requirement pre‐ and postpolymyxin B. Figure 1 depicts the temporal profile of the four patients with polymyxin‐associated neurotoxicity and the course of events in relation to the administration of polymyxin. On the *x*‐axis, D0 denotes the day of administration of polymyxin, prior to the loading dose (LD) of polymyxin. MD represents the maintenance dose, and the numbers 1 to 18 denote each of the doses. The arrows depict the point at which polymyxin level was sent in relation to the number of MDs, and when the dose of polymyxin was reduced or stopped. On the *y*‐axis, the four patients are represented as 1–4. The type of respiratory support that is provided in relation to the administration of polymyxin is denoted as being on room air (RA), nasal prongs (NP), venturi mask (VM), noninvasive ventilation (NIV) or invasive mechanical ventilation (IMV). In Patient 1, escalation of respiratory support from RA to NIV occurred with the LD. In Patient 2, escalation from RA to IMV occurred after the sixth MD. In Patient 3, patient went on from being on RA to requiring NIV after the first MD, whereas Patient 4 went on from being on NP to NIV after the fourth MD.

**Figure 2:**
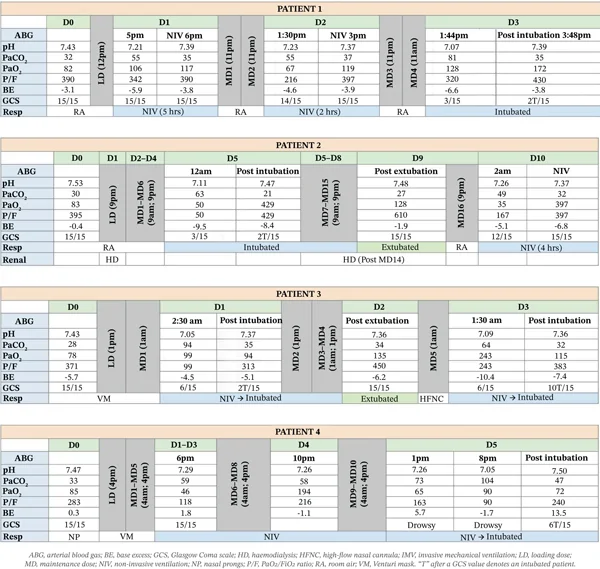
Arterial blood gases (ABGs) in Patients 1–4 pre‐ and postpolymyxin B. Figure 2 shows serial ABG estimation in the four patients, prior to polymyxin administration (D0) when the patient was either on room air (RA) or nasal prongs (NP). The ABGs on the subsequent days are represented as D1–D10. The relationship of the ABG with the type of respiratory support (Resp) is denoted at the bottom of each time period as noninvasive ventilation (NIV), intubated, or extubated. The Glasgow Coma Scale (GCS) at the time of sampling of ABG is also given; a score of T is assigned to the GCS when the patient is intubated, and the verbal score was not assessable. The number of maintenance doses (MDs) received prior to the ABG is also provided. Hemodialysis (HD) was required in one patient (Patient 2). PaO_2_ and PaCO_2_ are in mm Hg. BE indicates base excess.

In all the four patients, acute hypercapnic respiratory failure was temporally associated with polymyxin B infusions and resolved after dose adjustment or discontinuation (Figures 1 and 2). Brain imaging, in those in whom it was performed, did not reveal any abnormality that could contribute to central hypoventilation. There was no hemodynamic instability after the administration of polymyxin that could explain the onset or worsening of respiratory failure. Respiratory system examination and imaging did not reveal any pulmonary or pleural pathology to account for the respiratory failure. EMG/NCV studies, performed in Patient 1 after the ninth maintenance dose and in Patient 4 after the 18th maintenance dose, demonstrated sensory‐motor axonal polyneuropathy. However, given the timing of these studies after prolonged critical illness and mechanical ventilation, critical illness polyneuropathy cannot be excluded as an alternative explanation for these findings. Repetitive nerve stimulation studies, which may have better evaluated neuromuscular junction dysfunction, were not performed because polymyxin‐associated neurotoxicity was not initially suspected.

## 3. Discussion

This case series illustrates the possible association between polymyxin B administration and acute hypercapnic respiratory failure. The temporal association between polymyxin B administration and rapid progression of respiratory failure, together with improvement following drug discontinuation or dose reduction, suggests a possible association between polymyxin B and respiratory failure.

The WHO–UMC causality categories were assessed for each patient. Polymyxin has historically been associated with respiratory failure secondary to neuromuscular blockade. Patients 1 and 4 developed worsening acute Type II respiratory failure following polymyxin administration, with no alternative explanation identified. Improvement in ventilatory requirements after discontinuation of polymyxin, together with elevated plasma drug concentrations, supported a “probable” causal association according to the WHO–UMC causality assessment. Patients 2 and 3 additionally fulfilled the criteria for a positive rechallenge response, demonstrated by the recurrence of respiratory acidosis and the need for renewed ventilatory support following re‐exposure to polymyxin, with rapid improvement after drug withdrawal. Although the rechallenge was unintentional, the reproducible temporal relationship between drug re‐exposure and symptom recurrence, together with elevated plasma polymyxin concentrations and the absence of an alternative explanation, fulfilled the WHO–UMC criteria for a “certain” causal association.

Several mechanisms could have contributed to this phenomenon. In Patient 1, the onset of respiratory failure shortly after the loading dose, before significant drug accumulation would be expected, suggests a possible idiosyncratic neurotoxic reaction. In contrast, Patients 2–4 developed respiratory deterioration only after receiving multiple maintenance doses, a pattern that may be more compatible with cumulative or concentration‐associated neurotoxicity. Although polymyxin concentrations measured later during the clinical course were above the therapeutic range, the delayed timing of sampling limits conclusions regarding whether supratherapeutic concentrations were present at the time of acute respiratory deterioration. Individual susceptibility due to genetic polymorphisms, vulnerability of a critically ill patient, prior treatment with neurotoxic medications (e.g., chemotherapy), and batch‐related impurities could have contributed to the respiratory failure. None of the patients were receiving classical neurotoxic chemotherapeutic agents (e.g., vincristine, platinum compounds, or bortezomib) during the episode. Patient 3 had previously received azacytidine, which is not typically associated with peripheral neurotoxicity. Polymyxin‐associated neurotoxicity has been reported in 3%–17% of patients receiving systemic polymyxins [6]. The predominant reported symptoms included paresthesia, facial numbness, giddiness, ataxia, and seizures [6]. Polymyxin has been reported to disrupt neuron membrane integrity, inhibit acetylcholine release at the neuromuscular junction, and can induce biphasic neuromuscular blockade, which is initially competitive, followed by prolonged depolarization due to calcium depletion. This cascade can impair respiratory muscle function and explain the acute hypercapnic respiratory failure in our patients.

Two other factors could have contributed to neurotoxicity, namely infusion rate and dosing. Polymyxin B infusion is typically recommended over 1–4 h, with shorter 1‐h infusions potentially reducing renal toxicity by maximizing peak‐to‐trough variation. It is unclear if rapid infusion contributes to neurotoxicity. Infusion‐related reactions, including chest pain, dyspnoea, dizziness, and hypoxemia, have been reported with doses exceeding 200 mg per infusion [7]. In our series, the loading dose was 150 mg and maintenance doses ranged between 50 and 75 mg per infusion, all below the previously reported 200 mg threshold associated with infusion‐related reactions. Although this makes classical infusion‐related toxicity less likely, it cannot be completely excluded. Guidelines also state that polymyxin B does not require dose adjustment for renal impairment. This may be an oversimplification in critically ill patients with dynamic renal function and altered drug clearance [7], given that our study demonstrated supratherapeutic concentrations with the currently recommended doses.

Other factors could increase susceptibility to polymyxin neurotoxicity. Hypoxia, female gender, coadministration with neurotoxic antibiotics (e.g., aminoglycosides and sodium cephalothin), and concurrent use of neuromuscular blocking agents, sedatives, anaesthetics, narcotics, or corticosteroids were potential risk factors in other studies [8]. Experimental studies suggest that polymyxins given alongside glutamic acid to peripheral nerves can induce degenerative changes, supporting the drug′s direct neurotoxic potential [8].

Some limitations merit mention. Because four cases occurred over a short period, batch testing would have helped rule out drug impurities contributing to neurotoxicity. An important limitation is that polymyxin B concentrations were measured 3–8 days after the onset of respiratory failure rather than during the acute events themselves. Therefore, although concentrations were above the therapeutic range when eventually measured, these data cannot establish that supratherapeutic concentrations were present at the time of respiratory deterioration or definitively support a dose‐dependent toxicity mechanism.

The absence of repetitive nerve stimulation studies limits confirmation of neuromuscular junction dysfunction as the mechanism of respiratory failure.

Despite the above limitations, this series supports a possible link between polymyxin B and respiratory failure due to polymyxin‐induced neurotoxicity in critically ill patients. A prospective study is underway to assess the incidence and risk factors for polymyxin B–associated respiratory failure and explore likely mechanisms.

## 4. Conclusion

As antimicrobial resistance continues to rise—particularly in resource‐limited settings—the reliance on older agents such as polymyxin B has increased. However, this case series highlights the potential for acute, life‐threatening respiratory failure associated with its use. These findings underscore the importance of carefully weighing the risks and benefits when prescribing polymyxin B, even in the context of MDR Gram‐negative infections. Careful clinical surveillance is warranted in patients receiving polymyxin B, particularly those with prolonged therapy or altered drug clearance. Further research is needed to better define the mechanisms, risk factors, and optimal monitoring strategies for polymyxin‐associated respiratory failure.

## Funding

No funding was received for this manuscript.

## Disclosure

All authors qualify for full authorship.

## Conflicts of Interest

The authors declare no conflicts of interest.

## Data Availability

The data that support the findings of this study are available on request from the corresponding author. The data are not publicly available due to privacy or ethical restrictions.
